# The Posterior Unstable Shoulder: Natural History, Clinical Evaluation and Imaging

**DOI:** 10.2174/1874325001711010972

**Published:** 2017-08-31

**Authors:** Jorge Díaz Heredia, Miguel Angel Ruiz Iban, Raquel Ruiz Diaz, Santos Moros Marco, Juan Carlos Gutierrez Hernandez, Maria Valencia

**Affiliations:** 1 Unidad de Hombro y Codo. Servicio de Traumatología y Cirugía Ortopédica. Hospital Universitario Ramón y Cajal, Madrid, Spain; 2 Servicio de Traumatología y Cirugía Ortopédica, Clínica MAZ, Zaragoza, Spain; 3 Unidad de Hombro y Codo. Servicio de Traumatología y Cirugía Ortopédica. Fundación Jimenez Diaz, Madrid, Spain

**Keywords:** Posterior instability, Posterior luxation, Posterior subluxation, Posterior dislocation, Physical exam, Imaging

## Abstract

**Background:**

There is a low incidence of posterior instability which is present in only 2% to 10% of all unstable shoulders. The posterior instable shoulder includes different manifestations like fixed dislocation, recurrent subluxation or dislocation.

**Methods:**

Research and online content related to posterior instability is reviewed. Natural history, clinical evaluation and imaging are described.

**Results:**

An awareness of the disorder, together with a thoughtful evaluation, beginning with the clinical history, usually leads to proper diagnosis. An appropriate physical exam, taking in account hyperlaxity and specific tests for posterior instability should be done.

**Conclusion:**

Posterior shoulder instability is an uncommon condition and is challenging to diagnose. There is not a single injury that is responsible for all cases of recurrent shoulder dislocation or subluxation, and the presence of soft tissue lesions or bone alterations should be evaluated, with the use of adequate simple radiology and multiplanar imaging.

## 1 INTRODUCTION

The posterior unstable shoulder is an uncommon condition that is difficult to diagnose and is often unrecognized [1]. However, thanks to the increased awareness of the condition, incorrect diagnoses, delays in diagnosis, and even missed diagnoses are less frequent [2]. Usually different pathological changes can be found at the labrum, capsule, rotator interval, or bony architecture of the shoulder and often many of them coexist in the same patient [3].

Hippocrates was the first to describe the management of a patient with a lesion similar to a posterior acute dislocation. The first complete descriptions of this pathology were made by Sir Astley Cooper in 1839 in a patient with a seizure, and by Malgaigne in 1855, who presented a series of 37 patients with posterior instability [4].

The posterior unstable shoulder has different clinical presentation types. Patients can present a fixed acute or chronic posterior dislocation, recurrent posterior dislocation episodes or recurrent posterior subluxations [5]. Of these, recurrent posterior subluxations, which may present with instability symptoms, or without frank instability and only posterior shoulder pain, is the most common condition [6].

Glenohumeral instability affects approximately 2% of the general population. However there is a low incidence of posterior instability which is present in only 2% to 10% of all unstable shoulders [7]. Recurrent posterior subluxation is the most common type of posterior instability. Young men are the most affected group, and competitive athletes are a special risk group as both overuse injuries or single traumatic episodes can result in posterior subluxation or dislocation [4].

Although it may present as an isolated unidirectional problem, posterior instability frequently is found as a component of a complex shoulder laxity pattern combined with anterior dislocation or multidirectional instability. In this review the focus will be in isolated posterior instability.

## 2 AETIOLOGY

Typically the aetiology of posterior instability can be divided in two types: posterior instability of traumatic or atraumatic origin.

The traumatic origin might be due to a direct high-energy trauma or it can also develop insidiously through repetitive minor injury; these can also aggravate the symptoms of a patient with an initial major trauma [2]. A major trauma with a force applied to the arm with the shoulder in adduction, flexion and internal rotation is the most frequent cause [8] but seizures and electrocution that result in unbalanced contraction of the shoulder girdle muscles is also frequent. In adduction, internal rotation, and flexion, the pectoralis and latissimus dorsi muscles overpower the weak external rotators and cause internal rotation of the shoulder, displacing the humeral head superiorly and posteriorly against the acromion and medially against de glenoid fossa, resulting in posterior dislocation [8]. Other patients present a very different natural history: they have a history of minor trauma or repetitive microtrauma, and often have no history of true dislocations, but suffer symptomatic subluxations. This is due to progressive tissue damage as repetitive microtrauma or loading, like in throwing or over-head activities, promotes gradual posterior capsular failure.

True atraumatic case may be due to soft tissue abnormalities with capsular hiperlaxity, muscle patterning abnormalities, or glenohumeral dysplasia with high humeral and/or glenoid retroversion. Increased glenoid retroversion is a well-known predictor of recurrence and contralateral instability: Bradley *et al.* [9] reviewed 90 athletes surgically treated of posterior instability. On magnetic resonance arthrography, the shoulders with posterior instability were found to have significantly greater chondrolabral and osseous retroversion [10,7º +/- 3,^3^], in comparison with controls (5, 5º +/- 3,3). Gottschalk *et al.* [10] in a study with 28 patients, who suffered posterior dislocation, found that patients with retroversion of more than -16º showed a higher incidence of contralateral injuries.

Voluntary dislocation has to be taken in account. These types of patients are special because their shoulder problem might be related to attention-seeking behaviour and mental illness, and the treatment protocol should be different. However, the ability to dislocate the shoulder voluntarily is not entirely uncommon among people who are mentally healthy. Persistent voluntary posterior subluxation might initially do not generate discomfort for the patient, but it can gradually lead to pathologic changes in the capsule, causing pain and even involuntary posterior instability (voluntary dislocations that becomes involuntary) [11].

## 3 ANATOMY

The shoulder is the least stable joint in the body, and also the most mobile, because the humeral head is one third bigger than the glenoid fossa. Static and dynamic retainers constraints the humeral head against the glenoid fossa, but allows a large range of motion.

Static stabilization is collectively provided by the articular cartilage surfaces, glenoid labrum, capsular ligaments, and intra-articular pressure. In addition, glenoid version, humeral retroversion, and joint congruency help contribute to static stability [7]. Dynamic soft-tissue constraints are given by the rotator cuff, specially teres minor and subscapularis, the deltoid, the biceps tendon and also all the muscles involved in the scapulothoracic joint motion.

There is not a single injury that is responsible for all cases of recurrent shoulder dislocation or subluxation; however the posterior capsule, posterior band of the inferior glenohumeral ligament and posterior labrum provide the greater support posteriorly and are often involved. An isolated lesion of any of these posterior structures often results in unidirectional posterior instability.

## 4 PATHOLOGY

### Soft Tissue Lesions

The most common pathological finding associated with posterior shoulder instability is soft tissue injuries, like avulsion of the posterior labrum in his insertion in the glenoid associated with laxity of the inferior glenohumeral ligament [2].

A labral detachment at the postero-inferior part of the glenoid, with a tear in the periosteum, the so called reverse Bankart lesion, is usually seen in patients who have a prior history of a significant traumatic episode causing an acute posterior dislocation. The capsule-labral tear results in increased laxity of the posterior band of the inferior glenohumeral ligament and the posterior capsule; both enabling abnormal posterior translation of the humeral head.

Other patients develop a posterior labrocapsular periosteal sleeve avulsion (POLSPA) appears when the posterior labrum and the intact posterior scapular periosteum are stripped from the glenoid, producing a redundant recess [12].

Sometimes an extraarticular curvilinear calcification at the posteroinferior aspect of the glenoid can be observed, close to the posterior band of the inferior glenohumeral ligament; this is the Bennet lesion. It is usually found in throwing athletes, and is caused by repetitive strain to the ligament. Bennett lesions are frequently associated with tears of the posterior labrum in symptomatic shoulders [13].

Posterior labral tears have been classified by Kim *et al.* [14] into four types: Type I lesions are incomplete detachments of the labrum without displacement of the posteroinferior labrum. Type II are superficial tears between the posteroinferior labrum and the glenoid articular cartilage without complete detachment of the labrum [marginal crack], this is also called the Kim´s lesion. Type III are full chondrolabral erosions, these are quite similar to glenolabral articular disruptions (GLAD). Type IV is a flap tear of the posteroinferior labrum.

The humeral avulsion of the posterior band of the inferior glenohumeral ligament is called a reverse HAGL. The ligament detaches form the humeral side, and can be associated with an avulsion fracture of the posterior humerus at the insertion of the ligament [15]. Exceptionally a floating ligament can be found; in this condition a reverse HAGL is associated with a reverse Bankart lesion [16] or reverse bony bankart lesion [17].

Associated with these lesions or as an isolated entity, global laxity of the capsule can be present; this is usually evidenced during arthroscopy as capsular redundancy or an increased volume joint during magnetic resonance imaging with contrast [2]. This alteration of the elasticity of the collagen is present in patients with hiperlaxity with multidirectional instability or in patients with recurrent subluxation whose capsule undergoes plastic deformation.

### 4.1 Bone Lesions

There are different glenoid alterations that promote posterior shoulder instability like hypoplasia, higher retroversion, glenoid erosion or shear fracture.

Hypoplasia of the glena or humeral neck dysplasia are rare conditions [18] which often arise in bilateral manner and are usually associated with other tissue malformations. Pathogenically it is caused by an incomplete ossification of the lower two-thirds of the bony glenoid and scapular neck. It presents with an smooth and flat glenoid articular surface, and an abnormally hypertrophied posterior labrum. Posterior glenoid rim deficiency has been described as a hypoplasia located in the posterior region of the glenoid, there are two typical patterns: lazy J type or delta type [19]. This shape variation has been linked to cases of posterior instability [19, 20].

An excess of retroversion of the glena defined as more than 7º [21] in the sagittal plane, has been shown as a cause of increased posterior instability. Glenoid retroversion is best evaluated on axial CT, it is measured in the cut that is immediately inferior to the coracoid process. Normal glenoid retroversion is between - 2 ° and - 8 ° [3]. Gottschalk *et al.* [10] in a MRI study of patients who had suffered shoulder instability, associated a retroversion of the glena higher than 16 ° with higher risk of having an associated contralateral posterior dislocation.

Impaction lesions in the antero-superior area of the humeral head (reverse Hill-Sach lesions or McLaughlin lesions) are often found in patients with frank dislocations, even after the first episode; these lesions are more frequent in recurrent dislocation. This lesion is not a causative factor of first time dislocations but helps in the development of recurrent dislocations. These are present with varying sizes in up to 86% of the patients [22], with a significant lesion in 29% of the shoulders [23].

Occasionally a shear fracture of the posterior glenoid can be found, this is produced by the humeral head excursion during repetitive dislocations. It is known as a reverse bony Bankart lesion and can be seen in approximately 5% of the patients [23]. These lesions increase the recurrence rate after the first episode [1, 24].

More uncommon is the fracture of the lesser tuberosity by sharp contraction of the subscapularis muscle.

### 4.2 Clinical Presentation

Although posterior shoulder instability is uncommon, an awareness of the disorder, together with a thoughtful evaluation, beginning with the clinical history, usually leads to proper diagnosis. It is important to identify the first occurrence of instability as well as the first occurrence of shoulder pain. The exact location and timing of instability episodes, the nature of any behaviour that exacerbates the condition should also be determined.

Patients with an acute traumatic dislocation usually refer a history of trauma or seizure, although may be unable to provide adequate clinical history. On visual inspection, the shoulder often is in internal rotation with a prominent coracoid process and posterior fullness in the axilla. Physical examination may reveal a block to external rotation [8].

Patients with posterior instability, luxation or subluxation, typically present with a myriad of symptoms. They often report vague posterior-based shoulder discomfort that may or may not be associated with mechanical symptoms such as catching and clicking. Discomfort may be exacerbated by placing the arm in the provocative position, with the arm in 90º forward flexion, adduction and internal rotation. Activities that load the posterior aspect of the joint, such as the bench press or push-ups, often cause discomfort [2]. Athletes commonly report intensifying shoulder pain in the later stages of their sporting events when dynamic stability decreases due to muscle fatigue [3].

### 4.3 Physical Exam

An appropriate physical exam should begin with questioning the patient about his/her ability to voluntarily dislocate or subluxate their shoulder. Should the patient be willing to do that, this allows the examiner to identify the different positions of dislocation and assess the direction of the displacement.

Initially the complete active and passive range of motion should be evaluated. It is important that all the physical exam of the affected shoulder is compared to the contralateral shoulder. The scapulohumeral and scapulothoracic rhythm should be assessed to exclude the possibility of scapular winging, which is frequently confused with posterior instability [3].

To identify patients with hiperlaxity, the range of motion of other joints such as the elbow, wrist or first finger should be evaluated. The Gagey and sulcus tests are useful tools to evaluate laxity of the shoulder. To perform the sulcus test the patient sits down with the arm relaxed at side. The examiner centres the humeral head with a minimal compressive load and then stretches the arm downwards. The distance from the great tuberosity to the acromion should be address with the arm in 0º first and in 30º of external rotation afterwards. When, with the arm in external rotation, this distance is greater than 2 cm it is pathognomonic of hiperlaxity of the inferior ligament [25]. The Gagey test or hyperabduction test, is performed with the examiner holding down the scapula to prevent movement at the scapulthoracic joint, then the patient´s arm is abducted passively; If the arm is abducted over 105º there is significant inferior capsular laxity. A difference of more than 10º between sides is indicative of pathologic laxity. At present, this test has evolved to the “comparative hyperabduction test” in which both shoulders are examined and three items should be noted in order to consider the result positive. First, it has to reproduce patient´s deep pain; Secondly, it has to be asymmetrical when compared to the contralateral side [>20º] and third, a soft end point should not be felt [26].

The specific tests for diagnosis of posterior shoulder instability are the drawer test, Jerk test, Kim test and a reinterpretation of O´Brien test.

The Drawer test is performed with the patient in a sitting position with the arm at the side and the shoulder relaxed. The posterior drawer test is performed by holding the patient´s wrist or forearm [27] with one hand and placing the other hand over the patient´s shoulder so that the thumb is in the front and the fingers in the back. The examiner stabilizes the humeral head in a neutral position and applies a progressive force quantifying the anterior and posterior translation and the presence of clicking or subluxation. The arm should be forwardly flexed at the same time in order to allow the head to subluxate posteriorly. Saha *et al*. [28] described the “zero unpacked position” [arm elevated 45º to 60º] as the position in which shoulder has the most mobility. A 50% displacement of the humeral head is considered the upper limit of normal. It is not unusual to find symmetric posterior translation between the affected and unaffected shoulders [3]. A modification is the “load and shift” test that is performed with the patient supine and the affected shoulder at the edge of the examine table. The shoulder is positioned in the scapular plane in neutral rotation, and a posterior force is applied to assess the degree of translation of the humeral head.

The Jerk test is performed with the patient in a sitting position. While the examiner holds the scapula with one hand, the patient´s arm is abducted 90º and internally rotated 90º with the elbow flexed. An axial force is loaded with the examiner´s other hand holding the patient´s elbow, and a simultaneous horizontal adduction force was applied. A sharp pain with or without posterior clunk or click suggested a positive test result [29]. This test is more sensitive in detecting a predominantly posterior labral lesion. The Kim test is performed with the patient in a sitting position with the arm in 90º of abduction and the elbow flexed. With the examiner holding the patient´s elbow and lateral aspect of the proximal arm, a simultaneous axial loading force and 45 º upward diagonal elevations is applied to the distal arm, while inferior and posterior force is applied to the proximal arm. The presence of pain during the exploration with or without posterior clunk or click suggested a positive test [29]. This test is more sensitive in detecting a predominantly inferior labral lesion. The sensitivity in detecting a posteroinferior labral lesion increases to 97% when the Jerk test and the Kim test are combined [29, 30].

The O´Brien test is performed with the affected arm in 90º of forward flexion with the elbow in full extension. The patient then adducts the arm 10º to 15º medial to the sagittal plane of the body. The arm is internally rotated so that the thumb points downward. The examiner then applies a moderate downward force to the arm being resisted by the patient. This maneuver tightens the posterior capsule and posteriorly translates the humeral head; when there is a posterior labral injury cause abnormal loading of the rotator cuff with subsequent weakness. The test is considered positive when pain and objective weakness is observed by the examiner [31].

### 4.4 Imaging

#### 4.4.1 Simple Radiology

It is mandatory to obtain a true anteroposterior view in the scapular plane, a Y view and an axillary view to identify the normal bony anatomy, to ensure the joint is not dislocated, and to evaluate the presence of a reverse Hill-Sachs lesion or a Bennet sign. A West-point view usually is useful to detect osseous Bankart defects on the posterior glenoid rim.

#### 4.4.2 Multiplanar Imaging

Magnetic resonance imaging (MRI) is more useful in evaluating soft tissue pathology. It has a sensitivity of 90-94% for labral pathology [7]. The use of Intraarticular contrast [artro-MRI] improves the visualization of the posterior labrum and capsule, particularly with injuries such as capsulolabral disruptions or lateral capsular injuries [32].

Computed tomography (CT) is more useful to evaluate osseous pathology and glenoid and humeral orientation [7]. It is helpful to evaluate both glenoid shape and version, and also in defining the size and orientation of a reverse Hill-Sachs lesion, posterior glenoid bone loss, or bony Bankart lesion.

Functional electromyogram is also helpful to identify complex muscle pattern disorders, or in the diagnosis of braquial plexus injuries. Examination under anaesthesia and arthroscopy aid in the diagnosis and are key in defining the surgical procedure, although the surgeon should have most of the information beforehand.

## CONCLUSION

The awareness of the condition of the posterior instable shoulder, plus a meticulous exploration, including specific test for the posterior structures abnormalities, and also an adequate evaluation of the bone morphology and the soft tissue alterations would be the clue for correct diagnosis and the first step for an optimal treatment.
